# Staphylococcal biofilm formation on the surface of three different calcium phosphate bone grafts: a qualitative and quantitative in vivo analysis

**DOI:** 10.1007/s10856-015-5467-6

**Published:** 2015-02-19

**Authors:** Ulrika Furustrand Tafin, Bertrand Betrisey, Marc Bohner, Thomas Ilchmann, Andrej Trampuz, Martin Clauss

**Affiliations:** 1 Infectious Diseases Service, Department of Internal Medicine, University Hospital Lausanne (CHUV), Lausanne, Switzerland; 2 Unit of Septic Surgery, Department of Surgery and Anaesthesiology, University Hospital Lausanne (CHUV), Lausanne, Switzerland; 3 RMS Foundation, Bettlach, Switzerland; 4 Department for Orthopaedics and Trauma Surgery, Clinic for Orthopaedics and Trauma Surgery, Kantonsspital Baselland Liestal, Rheinstreet 26, 4410 Liestal, Switzerland; 5 Department of Traumatology and Reconstructive Surgery including Department of Orthopaedic Surgery, Charité Universitätsmedizin Berlin, Berlin, Germany

## Abstract

Differences in physico-chemical characteristics of bone grafts to fill bone defects have been demonstrated to influence in vitro bacterial biofilm formation. Aim of the study was to investigate in vivo staphylococcal biofilm formation on different calcium phosphate bone substitutes. A foreign-body guinea-pig infection model was used. Teflon cages prefilled with β-tricalcium phosphate, calcium-deficient hydroxyapatite, or dicalcium phosphate (DCP) scaffold were implanted subcutaneously. Scaffolds were infected with 2 × 10^3^ colony-forming unit of *Staphylococcus aureus* (two strains) or *S. epidermidis* and explanted after 3, 24 or 72 h of biofilm formation. Quantitative and qualitative biofilm analysis was performed by sonication followed by viable counts, and microcalorimetry, respectively. Independently of the material, *S. aureus* formed increasing amounts of biofilm on the surface of all scaffolds over time as determined by both methods. For *S. epidermidis*, the biofilm amount decreased over time, and no biofilm was detected by microcalorimetry on the DCP scaffolds after 72 h of infection. However, when using a higher *S. epidermidis* inoculum, increasing amounts of biofilm were formed on all scaffolds as determined by microcalorimetry. No significant variation in staphylococcal in vivo biofilm formation was observed between the different materials tested. This study highlights the importance of in vivo studies, in addition to in vitro studies, when investigating biofilm formation of bone grafts.

## Introduction

Infections associated with medical devices rarely occur, but represent a devastating complication with high morbidity and substantial costs [1]. Depending on the causing microorganism and host factors, these infections are typically caused by microorganisms growing in biofilms [1]. These microorganisms live clustered together in a highly hydrated extracellular matrix attached to a surface. Existence within a biofilm represents a basic survival mechanism by which microbes resist against external and internal environmental factors, such as antimicrobial agents and host immune system [2]. Depletion of metabolic substances and/or waste product accumulation in biofilms causes microbes to enter a slow- or non-growing state. Therefore, biofilm microorganisms are up to 1000 times more resistant to growth-dependent antimicrobial agents than their planktonic (free-living) counterparts [2–4]. For artificial joints and fracture-fixation devices the most common microorganisms causing infection are staphylococci [5, 6]. For prosthetic joint infection treatment is highly standardized [7] and eradication of infection is often only possible by removal of the implant and long-term antimicrobial treatment [8].

Bone transplantation is the most commonly performed transplantation, performed about 10-times more often than any other solid organ transplantation [9]. More than one million patients per year need a bone grafting procedure to repair a bone defect resulting from a trauma or a bone disease [10–12]. It is expected that bone grafts will be increasingly used in orthopaedic surgery to fill bone defects, and be used also as antimicrobial delivery systems [13]. The use of autologous cancellous bone grafts transplanted as fresh bone grafts is regarded as the gold standard [10, 14, 15]. However, several bone graft substitutes have been proposed, such as fresh-frozen allogeneic cancellous bone grafts [16, 17] and processed human or bovine cancellous bone grafts [18]. All these genuine bone grafts have a comparable calcium phosphate (CaP) architecture [11]. In the 1970s, various compositions of synthetic CaPs, such as β-tricalcium phosphate (β-TCP) or hydroxyapatite (HA), were proposed. Their importance and use have considerably increased over the past decades [19]. Besides differences in physico-chemical properties, resorption and osseointegration, artificial bone grafts differ in vitro in case of staphylococcal colonization and biofilm formation [11, 20]. As there is an increasing use of these bone substitutes, infections associated with these devices may also increase. While the “race to the surface” [6] as a multistep process of initial bacterial adhesion and later biofilm formation is well established for metal implants [6, 21–24] there is only limited data on in vitro [25, 26] and in vivo [27–29] biofilm formation on the surface of different CaP bone graft substitutes, mainly HA and TCP.

There are various methods for quantitative/qualitative evaluation of biofilm formation like “live-dead-staining” [30], confocal laser scanning microscopy [31], fluorescence microscopy [23, 25], electron microscopy (REM/SEM) [22, 23, 32] or atomic force microscopy (AFM) [32]. All methods need a special pre-treatment like staining (live-dead-staining, CFSM) or carbon-sputtering (REM/SEM) which hinder further biofilm investigation after quantification or might be impossible to assess on rough or 3D porous structures (AFM). In contrast, analysing biofilm formation on the surface of various porous materials by means of sonication and microcalorimetry has been shown to be a robust test not necessitating a pre-treatment of the biofilm in vitro [11, 20, 33].

In a recent in vitro study, we investigated by sonication and microcalorimetry biofilm formation on the surface of three different but morphologically similar CaPs, β-TCP (cyclOS), dicalcium phosphate (DCP) and calcium-deficient HA (CDHA). We were able to demonstrate a lower amount of biofilm on the β-TCP, compared to the DCP and the CDHA. As the in vitro setting is very different from the clinical situation we wanted, as a next step, to confirm our findings in an in vivo setting. The aim of this study was to investigate in vivo biofilm formation on the surface of three well characterized CaP bone grafts [β-TCP (cyclOS), DCP, CDHA], and to compare the results to the in vitro data [33].

## Materials and methods

### Bone grafts

Three different CaP bone grafts [β-TCP (cyclOS), DCP, CDHA], with recently published physico-chemical characteristic [20] were used (Table 1). Samples were obtained as sterilized cylinders (6.5 × 10 mm).

**Table 1:** Summary of the physico-chemical properties of the samples used in the present study

Materials	Compositions	Specific surface area (m^2^/g)	Macropore diameter (mm)	Apparent density (g/cm^3^)	Porosity (%)	Porosity^a^ (%)	Porosity accessible by bacteria (>1.5 μm)^a^ (%)	d_50_^a^ (μm)
β-TCP (cyclOS)	>99 %^b^	0.84 ± 0.15	0.26 ± 0.07	0.88 ± 0.03	71.1 ± 1.0	70 ± 3	59 ± 3	17 ± 3
DCP	93 % DCP, 6 % α-TCP, 1 % DCPD	4.04 ± 0.35	0.37 ± 0.08	1.17 ± 0.04	60.0 ± 1.4	46 ± 2	37 ± 2	27 ± 7
CDHA	98 % HA, 2 % DCP	43.6 ± 0.4	0.53 ± 0.13	0.53 ± 0.01	82.0 ± 0.3	69 ± 4	27 ± 9	0.23 ± 0.18

Crystalline composition (Rietveld refinement analysis of the XRD data), specific surface area (SSA), macropore diameter, apparent density, porosity, median pore size (d_50_) and porosity accessible by bacteria (>1.5 μm) in mean and standard deviation (from [20])

^a^Determined by mercury porosimetry

^b^Crystallite size 103 ± 12 nm (±1 St Dev)

### Study organism

Two *S. aureus* strains (ATCC 29213, methicillin-susceptible and ATCC 43300, methicillin-resistant) [34] and one *S. epidermidis* strain RP62A (ATCC 35984, methicillin-susceptible) [33] were used. The strains were stored at −70 °C using a cryovial bead preservation system (Microbank, Pro-Lab Diagnostics, Richmond Hill, Ontario, Canada). For preparation of the inoculum, a single bead was freshly grown on sheep blood agar overnight. Bacterial inocula were prepared from discrete colonies resuspended in sterile 0.9 % saline (NaCl) to a McFarland turbidity of 0.5 representing a bacterial concentration of ~1.0 × 10^7^ colony-forming units (CFU)/mL. The stock solution was diluted 1:1000 for further experiments.

### Animal model

An established foreign-body infection model in albino guinea pigs was used [35, 36]. The guinea pigs were kept in the Animal House of the University Hospital Lausanne and animal experimentation guidelines according to the regulations of Swiss veterinary law were followed. The study protocol was approved by the Institutional Ethical Committee. In brief, four sterile polytetrafluoroethylene (Teflon) cages (32 mm × 10 mm) perforated with 130 regularly spaced holes of 1 mm in diameter (Angst-Pfister AG, Zurich, Switzerland) prefilled with one CaP scaffold were subcutaneously implanted in the flanks of male albino guinea pigs (Charles River, Sulzfeld, Germany) under aseptic conditions. Animals weighing 550–600 g were anesthetized with subcutaneous injection of ketamine (20 mg/kg of body weight) and xylazine (4 mg/kg). Two weeks after surgery and healing of the surgical wounds, interstitial fluid accumulating in tissue cages was checked for sterility. Contaminated cages were excluded from further experiments. Experiments were performed in two animals in parallel carrying the same CaP scaffold in all four tissue cages (i.e., eight replicates per material). On day 0, three out of four tissue cages/animal were infected by inoculating 2 × 10^3^ CFU/cage of either *S. aureus* ATCC 29213 (MSSA), *S. aureus* ATCC 43300 (MRSA) or *S. epidermidis* RP62A ATCC 35984 (MSSE) with a sterile syringe. The fourth uninfected cage served as negative control. Animals were infected for 3, 24 and 72 h, respectively, according to an established in vitro setting [11]. Afterwards animals were killed by toxic CO_2_ and CaP samples with the surrounding cage were harvested in the animal house after disinfection of the skin and stored in 50 mL Falcon tubes prefilled with 5 mL 1 % of phosphate buffered saline (PBS) for biofilm analysis (see hereafter).

### Biofilm analysis

Biofilm analysis was performed under laminar flow and adapted from our recently published procedure [33] including three steps (i) harvesting of the scaffolds and washing procedure, (ii) sonication and (iii) a final microcalorimetric analysis.

#### Harvesting of the scaffolds and washing procedure

**Fig. 1:**
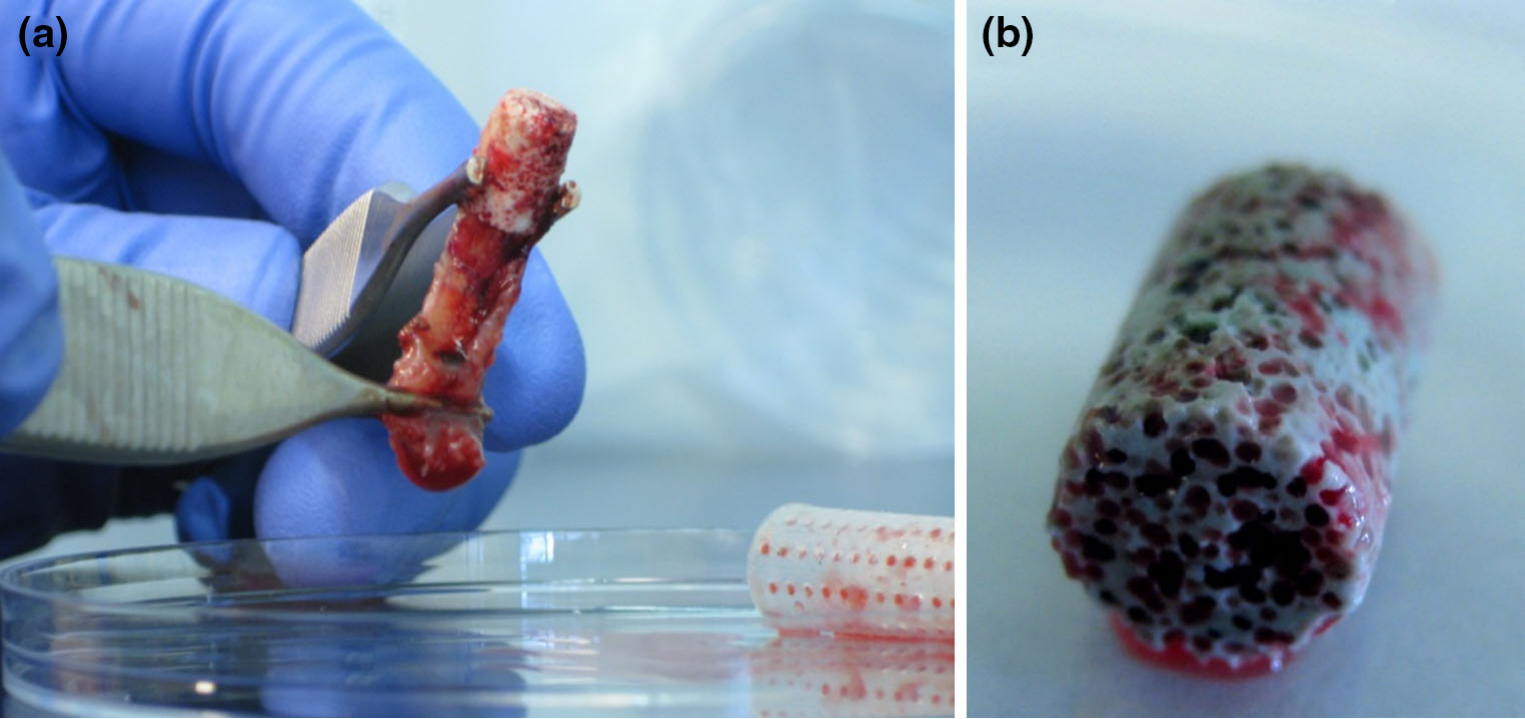
**a**, **b** CaP scaffold with surrounding soft tissue envelope explanted from the cage (*right corner*) and after peeling off soft tissue

After harvesting of the scaffolds further processing was done under laminar flow in the microbiology laboratory. CaP scaffolds were transferred to a new 50 mL-Falcon tube (prefilled with 5 mL PBS) with a sterile forceps after peeling of the surrounding soft tissue envelope (Fig. 1a, b). They were carefully washed five times with 5 mL 1 % PBS to remove planktonic bacteria. For washing the PBS was poured in the Falcon tubes by placing a glass pipette on the wall of the Falcon tubes, afterwards the Falcon tubes were shaken cautiously by hand and in a final step the PBS was aspirated by placing a Pasteur pipette atop one side of the CaP scaffolds to have a flush through the scaffold. Both the glass pipette and the Pasteur pipette were changed after processing one scaffold to avoid contamination from one sample to another.

#### Sonication procedure

After washing, samples were transferred to new 50 mL-Falcon tube containing 5 mL PBS, gently shaken for 10 s, sonicated at 40 kHz for 1 min in a bath tub sonicator (BactoSonic, Bandelin, Germany) and shaken again for 10 s. The dislodged biofilm (sonication fluid) was transferred to a 2 mL Eppendorf tube and CaP bone grafts were stored for microcalorimetry (see hereafter).

Sonication fluid was serially diluted in Eppendorf tubes and aliquots of 100 µL were plated on sheep blood agar and incubated at 37 °C aerobically for 24 h. Bacterial counts were enumerated and expressed as CFU/sample. Plates were rated countable between 1 and 500 CFU/plate and examined for variations in colony morphology (colour, size) and contaminations.

#### Microcalorimetry protocol

All microcalorimetry tests were performed using a 48-channel batch calorimeter (thermal activity monitor, model 3102 TAM III; TA Instruments, New Castle, DE, USA).

In more details, CaP samples were transferred into sterile 4 mL calorimeter ampoules pre-filled with 1 mL of tryptic soy broth, closed with a rubber cap and sealed by manual crimping. Ampoules were sequentially introduced into the microcalorimeter and remained 15 min in the thermal equilibration position before lowering into the measurement position. Heat flow was measured continuously after the signal stability was achieved throughout an 18 h-period and expressed as heat flow over time [in microwatts (µW)]. The calorimetric time to detection (TTD) was defined as the time from insertion of the ampoule into the calorimeter until the exponentially rising heat flow signal exceeded 20 µW to distinguish microbial heat production from the thermal background. TTD indirectly quantifies the amount of bacteria with a shorter TTD representing a higher amount of bacteria. Data analysis was performed by the manufacturer’s software (TAM Assistant; TA Instruments) and Prism 5.0 (GraphPad Software, La Jolla, CA).

### Statistical calculations

To equalize variances in bacterial counts, data are presented as log_10_ CFU/sample. For statistical analysis one-way ANOVA with Tukey’s multiple comparison test was performed using Prism 5.0 (GraphPad Software, La Jolla, CA). A *P* value <0.05 was considered to be significant.

## Results

During the experiments none of the animals showed systemic signs of infection (i.e., all infections remained local) and all animals showed the expected weight gaining over time representing animal welfare. Uninfected CaP scaffolds used as negative experimental controls remained sterile throughout the experiment.

**Fig. 2:**
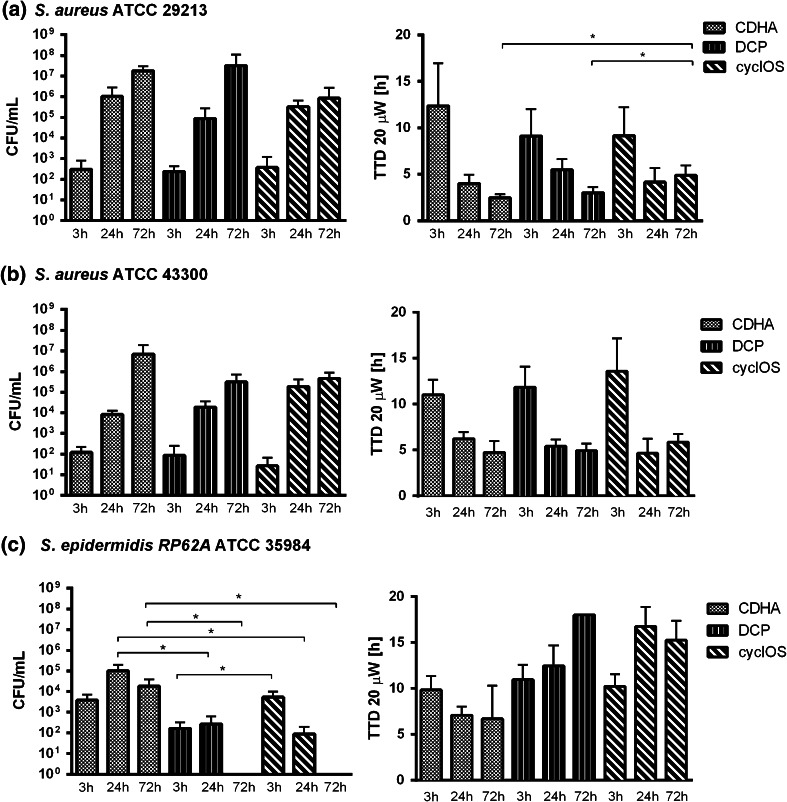
*S. aureus* ATCC 29213 (**a**), *S. aureus* ATCC 43300 (**b**) and *S. epidermidis* RP62A (**c**) bacterial counts in sonication fluid (*left*) compared with microcalorimetry results (*right*). * *P* < 0.05

*Staphylococcus aureus* ATCC 29213 and ATCC 43300 formed an increasing amount of biofilm on the surface of all scaffolds over time (Fig. 2a, b). For both *S. aureus* strains a statistically significant (*P* < 0.05) increase was observed between 3 and 24 h, and 3 and 72 h of infection, respectively, on the three materials. There was no significant further increase in amount of biofilm between 24 and 72 h of infection. When comparing the three materials, significantly less biofilm of *S. aureus* ATCC 29213 was detected by microcalorimetry on cyclOS compared to DCP and CDHA after 72 h of infection (*P* < 0.05). However, no statistical difference between the materials was observed by sonication and viable count. For *S. aureus* ATCC 43300, there were no statistical differences between the three materials at any time point.

For *S. epidermidis* RP62A the results were less homogeneous and a decrease in biofilm amount over time was observed for DCP and cyclOS (Fig. 2c). Sonication (CFU/mL, left panel) showed a heterogeneous picture with an increase amount of biofilm on the CDHA and DCP scaffolds but a decreasing amount on the cyclOS scaffolds (not statistically significant) between 3 and 24 h after inoculation. Furthermore, 72 h after inoculation, sonication showed no biofilm on the surface of the DCP and cyclOS scaffolds indicating a clearing of the infection, whereas a stable amount of biofilm was detected on the CDHA scaffolds. At all time points, significantly less biofilm was found on DCP compared to CDHA (*P* < 0.05) by sonication and viable count. After 24 and 72 h of infection, significantly less biofilm was also found on cyclOS compared to CDHA (*P* < 0.05). Less biofilm was found on DCP compared to cyclOS after 3 h of infection (*P* < 0.05). Microcalorimetry (right panel) showed a stable amount of biofilm on the CDHA scaffolds over time. For DCP there was a stable amount of biofilm between 3 and 24 h after inoculation but a clearing of the infection after 72 h incubation (TTD >18 h). Both findings were in concordance with results obtained by sonication. On the cyclOS scaffolds there was a decrease in biofilm amount between 3 and 24 h (*P* < 0.05) after inoculation, which was in concordance with sonication. 72 h after inoculation microcalorimetry showed less biofilm on cyclOS as compared to 3 h (*P* < 0.05) but more biofilm as compared to 24 h after inoculation (not statistically significant). As observed by sonication and viable counts, less biofilm was observed on DCP and cyclOS compared to CDHA after 24 and 72 h of infection (*P* < 0.05). In addition, less biofilm was observed on cyclOS compared to DCP after 24 h of infection.

**Fig. 3:**
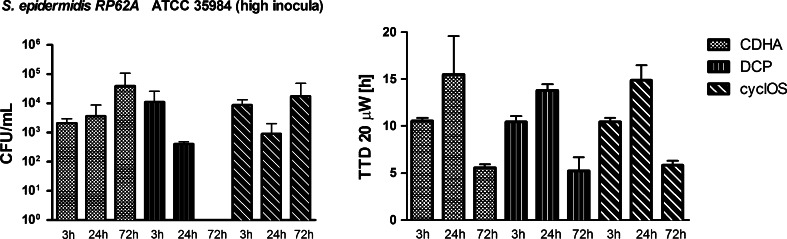
Results from additional experiments with a higher infection inoculum of *S. epidermidis*

Additional experiments with *S. epidermidis* RP62A with a higher initial inoculum (from 1 × 10^5^ to 1 × 10^7^ CFU) were performed to investigate whether clearing of the infection with the DCP and cyclOS scaffolds was due to the material or if the initial inoculum had been too low to establish a stable biofilm infection. With the higher inoculum, the infection remained stable on all scaffolds but the amount of bacteria found on the scaffolds by sonication varied between the materials (Fig. 3, left panel). By sonication, no bacteria could be dislodged from three of three scaffolds for DCP, and in two of three scaffolds for CDHA and cyclOS after 72 h of infection. In contrast, microcalorimetry showed the shortest TTD at 72 h for all three tested materials (Fig. 3, right panel). When comparing the three different materials, no significant differences in biofilm formation was observed at any time point.

## Discussion

Staphylococcal foreign-body infection is a significant complication for orthopaedic patients undergoing surgery, particularly with fracture fixation and arthroplasty. Given the difficulty in studying *S. aureus* infections in human subjects, animal models serve an integral role in exploring the pathogenesis of these infections, and aid in determining the efficacy of prophylactic and therapeutic treatments. Animal models should mimic the clinical scenarios seen in patients as closely as possible to permit the experimental results to be translated to the corresponding clinical care.

There is no animal model which is defined as the gold standard for the investigation of staphylococcal biofilm formation but the course of a foreign-body infection in the guinea pig model is similar to that observed in humans [37], and thus the guinea-pig model might come closest to such a definition. In contrast to mice and rats no spontaneous cure of infected implants occurs [45]. As we expected small differences between the materials, all experiments were performed with a relatively small starting bacterial inoculum (2 × 10^3^ CFU/cage) as compared to other experiments using the same strains using inocula of 104–107 CFU/cage [34, 38–40].

We obtained a stable infection for both *S. aureus* strains on the surface of all CaP scaffolds. Interestingly the amount of biofilm was always lower for the MRSA (ATCC 43300) as compared to the MSSA (ATCC 29213) strain. Even though differences were small this observation might represent the reduced “fitness” of the MRSA strain which can also be seen in the clinical situation. As observed in our in vitro study [20], less MSSA biofilm was observed on cyclOS compared to DCP and CDHA. However, in the in vivo setting this could only be observed by microcalorimetry after 72 h of infection.

Our results obtained with the low inoculum of *S. epidermidis* were conflicting, when compared to in vitro results showing reduced biofilm formation on cyclOS using the same materials and methods [20]. In the in vivo setting less biofilm was detected on both DCP and cyclOS in comparison to CDHA, and in addition less biofilm was dislodged from DCP compared to cyclOS. When using an infection inoculum of 2 × 10^3^, we observed a spontaneous clearing of the infection 72 h on after inoculation of the DCP material (e.g., no biofilm could be detected by viable counts or sonication). Widmer et al. [40] did not observe any spontaneous cure of *S. epidermidis* infection with a starting inoculum of 10^4^ CFU/cage using the same animal model. Thus it remains unclear whether a stable infection could be established in the cage or whether DCP is resistant to *S. epidermidis* biofilm formation with the low inoculum used. With the higher inoculum, microcalorimetry showed comparable amounts of biofilm formation on the surface of all CaP scaffolds, suggesting that a stable infection cannot be established using a low infection inoculum on the DCP material. DCP is considered to be acidic compared to β-TCP or CDHA because it contains HPO_4_ groups instead of PO_4_ groups [41]. Once present in the body, DCP can theoretically convert to CDHA or HA releasing acidic components (phosphoric acid) which might interfere with bacterial growth.

Furthermore sonication and viable count showed a clearance of the infection on the DCP after 72 h of infection even with the higher inoculum. This, in comparison to microcalorimetry, contradictory result could be explained by the higher sensitivity of the microcalorimeter. Whereas the sonication allows quantification of detached biofilm bacteria through viable count, the microcalorimeter measures the bacterial presence on and within the scaffold during 18 h in a rich culture media allowing detection of small bacterial quantities as well as dormant bacteria.

In a recent in vitro study, we investigated by sonication and microcalorimetry biofilm formation on the surface of morphologically similar CaPs. We found that biofilm formation was comparable for CDHA and DCP, but lower for cyclOS [20]. These in vitro results suggested that biofilm formation was not influenced by a single physico-chemical parameter alone but is a multi-step process influenced by several factors in parallel. Adherence to the surface involves nonspecific physical factors (e.g., surface tension, hydrophobicity, and electrostatic interaction) and specific bacterial and host adhesins such as fibronectin. This initial process is followed by biofilm formation, which is mediated in part by the polysaccharide intercellular adhesion (ica) encoded by the *ica* operon [42]. While in the in vitro setting bacteria were added to the CaP scaffolds after 30 min of incubation in human serum [20], the time between implantation of the CaP scaffold and bacterial inoculation in the in vivo setting was 14 days. A 2-week long period is needed in order to allow complete wound healing after surgery. The wound healing is especially important for animal welfare but also for avoiding contamination of the implants during manipulation of the animals. Due to this prolonged time period protein adsorption on the surface of the CaP scaffolds was significantly different between the in vitro and in vivo setting. Whereas biofilm formation in an in vitro setting only is influenced by nonspecific physico-chemical factors, the in vivo setting includes the interaction between bacteria and adhesins, especially fibronectin, covering the implant surface. In other words, the physico-chemical differences are enveloped leaving only the macroscopic texture of the CaP scaffolds which is rather comparable [20] explaining the minor experimental differences between the materials in vivo. In order to be closer to the in vitro setting, another animal model using pre-infected implants, such as the rat model presented by Monzon et al. [43], could have been used. In a clinical situation, bone grafts may be infected either during surgery or post-operative due to disturbed wound healing [44]. With post-operative contamination tissue integration of the bone graft has already started and the tissue-cage model used in this study might be more representative for the clinical problem [44]. Another limitation of the study was that the animal model used did not include local factors generated during bone integration as the CaP scaffolds were implanted subcutaneously and not directly into the bone.

## Conclusion

Whereas, significantly less mature MSSA in vivo biofilm could be observed on cyclOS compared to CDHA and DCP, no significant variation in MRSA in vivo biofilm formation was observed between the different materials tested. With a low inoculum of *S. epidermidis* we found less biofilm on DCP and cyclOS compared to CDHA, and a clearance of the infection on the DCP bone grafts was observed which might be explained by the release of HPO_4_. The experimental setting represents an in vivo post-operative contamination model suitable to study the race-to-the-surface. This study highlights the importance of considering in vivo factors when investigating biofilm formation of bone grafts.
